# Alternation of the gut microbiota in irritable bowel syndrome: an integrated analysis based on multicenter amplicon sequencing data

**DOI:** 10.1186/s12967-023-03953-7

**Published:** 2023-02-11

**Authors:** Han Chen, Rong Ou, Nana Tang, Wei Su, Ruoyun Yang, Xin Yu, Guoxin Zhang, Jianhua Jiao, Xiaoying Zhou

**Affiliations:** 1 Department of Gastroenterology, The First Affiliated Hospital of Nanjing Medical University, 300# Guangzhou Road, Nanjing, 210029 People’s Republic of China; 2 The First Clinical Medical College, Nanjing Medical University, Nanjing, China; 3 Department of Gastroenterology and Hepatology, Jinhu County People’s Hospital, Huaian, China

**Keywords:** Irritable bowel syndrome, Gut microbiota, Amplicon sequencing analysis, Propensity score matching, GMrepo database

## Abstract

**Background:**

Gut dysbacteriosis has been reported as one of the etiologies for irritable bowel syndrome (IBS). However, the association between gut microbiota and IBS is still inconclusive.

**Method:**

A paired-sample study was designed by retrieving original multicenter 16 s-rRNA data of IBS patients and healthy controls from the GMrepo database. The propensity score matching (PSM) algorithm was applied to reduce confounding bias. The differential analysis of microbiota composition was performed at different taxonomic levels. The co-occurrence network was established. Subgroup analysis was performed to identify specific microbial compositions in different IBS subtypes.

**Results:**

A total of 1522 amplicon samples were initially enrolled. After PSM, 708 individuals (354 IBS and 354 healthy controls) were eligible for further analysis. A total of 1,160 genera were identified. We identified significantly changed taxa in IBS groups (IBS-enriched: the families *Enterobacteriaceae*, *Moraxellaceae* and *Sphingobacteriaceae*; the genera *Streptococcus*, *Bacillus*, *Enterocloster*, *Sphingobacterium*, *Holdemania* and *Acinetobacter*. IBS-depleted: the phyla *Firmicutes*, *Euryarchaeota*, *Cyanobacteria*, *Acidobacteria* and *Lentisphaerae*; the families *Bifidobacteriaceae*, *Ruminococcaceae*, *Methanobacteriaceae* and the other 25 families; the genera *Faecalibacterium*, *Bifidobacterium* and other 68 genera). The co-occurrence network identified three hub genera and six hub species (including *Faecalibacterium prausnitzii*) that may be involved in IBS pathophysiology. Strong positive interactions were identified among the *Bifidobacterium longum*, *Bifidobacterium breve* and *Bifidobacterium adolescentis* in the *Bifidobacterium* community.

**Conclusion:**

This study provides quantitative analysis and visualization of the interaction between the gut microbiota and IBS. The identification of key species should be further validated to evaluate their causal relationships with the pathogenesis of IBS.

**Supplementary Information:**

The online version contains supplementary material available at 10.1186/s12967-023-03953-7.

## Introduction

Irritable bowel syndrome (IBS) is a commonly encountered functional gastrointestinal disorder with a relatively high prevalence of 7–16% population and an estimated annual medical cost of more than $1 billion in the United States [1]. For decades, the pathophysiology of IBS has not been well explained. Various factors may be involved in the pathogenesis of IBS, including impaired gut-brain interactions, gut microbiome dysbiosis, altered GI motility and abnormal visceral sensation [2, 3].

In recent years, advances in 16S rRNA and metagenome sequencing technologies have facilitated the exploration of the gut microbiota composition in patients with IBS [4]. Several cross-sectional or case-control studies [5–10] have demonstrated that IBS patients exhibit a reduction in beneficial commensal genera compared with healthy individuals. However, microbial compositions in IBS individuals are still inconsistent in different studies, probably due to various methodologies they applied to identify microbes for distinguishing IBS and healthy controls. Thus, the taxonomic change of IBS on specific microbes is still conflicting and inconclusive [11]. Furthermore, most relevant studies were single-center with small sample sizes. Several studies [7–10] even failed to establish a convincing case-control comparison due to the lack of specific individual information, such as sex and age. Therefore, multicenter microbial data with relatively large sample sizes are still needed to clarify the microbial change in the pathophysiology of IBS. In addition, fecal microbiota transplant (FMT) may relieve the symptoms in patients with IBS by improving microbial dysbiosis. However, the therapeutic efficacy of FMT is also controversial based on the contradictory results reported from different studies [12, 13].

Therefore, we designed a paired-sample microbial analysis by integrating the 16 s rRNA sequencing data of IBS and healthy controls from multicenter studies in the GMrepo database [14]. This study will clarify a more precise microbiota panel in IBS by providing an updated quantitative interpretation of microbial composition differences between IBS and healthy individuals and exhibiting a visualization of the interaction of different microbial taxa in IBS patients. It will also provide new evidence for future FMT in IBS patients.

## Methods

### Study design and data collection

This study enrolled microbial sequence data from human stool samples with IBS (Medical Subject Headings (MeSH) Unique ID: D043183) and health controls (MeSH Unique ID: D006262). The 16 s rRNA sequencing data from human stool samples were retrieved from the GMrepo database (https://gmrepo.humangut.info), an easily-accessed online database facilitating the accessibility of the rapidly growing human microbial data [15]. This database provides microbiota data from different study projects with personal information on age, sex, region and body mass index (BMI). We included eligible sequence data of patients: (1) diagnosed with IBS; (2) aged more than 18 years and with body-mass-index (BMI) of more than 15; (3) with positive quality control (QC) status in the database; (4) with accessible amplicon sequence data (16S rRNA gene sequence). Exclusion criteria include: (1) a recent history of antibiotics use; (2) a sample with missing information of sex, region, age, or BMI. (3) IBS combined with inflammatory bowel disease (IBD), severe intestine infection, or other chronic infectious diseases. The sub-types of IBS were determined by extracting the supplemental information of patients in the database. IBS-C was defined as IBS complicated with predominant constipation and IBS-D as IBS complicated with predominant diarrhea. IBS with psychological disorders were defined as IBS patients accompanied by at least one of the following disorders: Migraine Disorders, Autism Spectrum Disorder (ASD), Depression, Bipolar Disorder, Schizophrenia and Attention Deficit Disorder with Hyperactivity.

### The PSM procedure

To reduce confounding bias, a propensity score matching (PSM) algorithm was applied to generate cohorts of IBS and their matched healthy individuals by controlling age, sex, region and BMI using SPSS Statistics for Windows, Version 25.0 (IBM) Corporation, Armonk, NY) [16]. The match ratio of patients in both groups was 1:1, with 0.01 match tolerance.

### Statistical analysis of the baseline characteristics

Statistical analysis was performed using R software (version 4.1.0). Normality tests were applied by the Shapiro-Wilk test. Data with normal distribution were considered as p-value > 0.05 and are presented as mean and standard deviation, and data with non-normal distribution are presented as median with Interquartile Range (Q). For comparisons, the student *t*-test was applied for data with normal distribution, and the Wilcoxon Rank Sum Test was performed in independent data with non-normal distribution. Pearson chi-square or Fisher’s exact test was applied to compare categorical variables. The R Packages of MatchIt, optmatch and reshape2 were used to generate matched cohorts through the PSM algorithm.

### Data processing, quality control and taxonomic classification

FastQC (version 0.11.8) was applied to estimate the overall quality of the downloaded data, followed by the use of trimmomatic to remove vector sequences and low-quality bases. Microbial sequences shorter than 2/3 of the length of the original reads were removed from subsequent analysis. The remaining sequences were considered qualified clean data and were used for subsequent analysis. As for the steps of quality *control, S*amples (runs) with less than 20,000 clean reads were first removed from the subsequent analysis and marked as “failed QC (QC status = 0)”. Then, after taxonomy assignment, samples containing only a single taxon (i.e., a species or genus that accounts for more than 99.99 percent of total abundance) will be marked as “failed QC”. For whole genome (i.e., metagenomic or mNGS) sequences, MetaPhlAn2 was used with default parameters for the taxonomic classification of the sequencing reads. Each data of relative abundance in every sample was collected and integrated into a merged taxonomic abundance table. NCBI taxonomy database was searched for classifying organisms in different levels (Kingdom, Phylum, Class, Order, Family, Genus, Species). The taxonomic composition of each sample was integrated into a final taxonomy classification table.

### The alpha and beta diversity

The diversity of the microbiome in the human data was evaluated by R software (version 4.1.0, http://www.R-project.org/). Alpha diversity was evaluated by the Shannon index, Simpson index and richness (number of observed species), using the “vegan” package in R. The difference in alpha diversity was calculated by Wilcoxon rank-sum tests. Beta-diversity was presented by unconstrained principal coordinate analyses (PCoA) scatter plots via calculating Bray-Curtis distances. Permutational multivariate analysis of variance (PERMANOVA) was then used to determine the difference between different phenotypes [17].

### Differential analyses of microbial compositions

The pairwise differential analysis was completed using the Wilcox.test in R. Multiple comparisons were performed using the Kruskal.test in R. Manhattan plot was generated using packages of metagenomeSeq and ggplot2. The Spearman correlation analysis was performed and visualized in R using the package corrplot, ggcor and ggplot2. Clustering analysis with Z-score standardized data was applied to visualize the component difference of microbial species in the IBS and control groups, using the pheatmap package in R. The plot of clustering analysis, Venn and species distribution in different subgroups were completed using the package “ggvenn” for Venn and package “ggplot2”, “tidyverse” and “reshape2” for species distribution. Microbial interactions with age or BMI were investigated using the Spearman correlation. The adjusted p-values generated from multiple comparisons were also calculated using the false discovery rate (FDR) method.

### Co-occurrence network

A correlation matrix was constructed by calculating the pairwise Spearman’s rank correlations in all IBS samples. A correlation between two microbes was considered statistically robust if Spearman’s correlation coefficient was > 0.8 and the *p*-value was < 0.01 [18]. To reduce the chances of obtaining false-positive results, the *p*-values were adjusted using the Benjamini–Hochberg method. The molecular ecological network analyses (MENA) were applied to construct random matrix theory (RMT) based on co-occurrence bacterial networks and presented in Cytoscape Version 3.9.1. The most densely connected node was defined as the hub microbe, and hub microbes were identified using the cytohubba module.

### Identification of microbial biomarker for IBS

Finally, a random forest model [19] was built using R package randomForest. Significant microorganisms were incorporated into a panel for classifying IBS, at the genus and species levels, respectively.

## Results

### General characteristics of the study samples

Following the study criteria, 1522 amplicon samples (365 IBS and 1157 health controls) were identified. Before the PSM procedure, there were significant distribution differences in region (p < 0.001), sex (p < 0.001) and BMI (*p* = 0.027) between IBS and control groups. After PSM, the age, sex, region and BMI showed no statistical difference between IBS and control groups. A total of 708 samples (354 IBS and 354 health controls) were enrolled in the final analysis. The baseline characteristics are shown in Table 1 and Additional file 1: Table S1.

**Table 1:** Baseline characteristics of patients before and after propensity score matching

No. of patients	Before match			After match		
	IBS (n = 365)	Controls (n = 1157)	*p*	IBS (n = 354)	Controls (n = 354)	*p*
Age (median [IQR])†	45 [33, 59]	45 [35, 57]	*0.407*	45 [36, 57]	46 [35, 59]	*0.610*
BMI (median [IQR])†	23.67 [21.46, 25.80]	22.80 [20.94, 5.62]	0.027*	22.90 [20.97, 25.65]	23.03 [20.98, 25.48]	*0.605*
Country (n, %)			< 0.001*			*1.000*
USA	175 (47.95)	832 (71.91)		175 (49.44)	175 (49.44)	
UK	169 (46.30)	274 (23.68)		160 (45.20)	160 (45.20)	
Australia	11 (3.01)	19 (1.64)		9 (2.54)	9 (2.54)	
Switzerland	6 (1.64)	12 (1.04)		6 (1.69)	6 (1.69)	
Canada	4 (1.10)	20 (1.73)		4 (1.13)	4 (1.13)	
Sex (n, %)			< 0.001*			*1.000*
Female	238 (65.21)	488 (42.18)		227 (64.12)	227 (64.12)	
Male	127 (34.79)	669 (57.82)		127 (35.88)	127 (35.88)	

*IBS* irritable bowel syndrome, *BMI* body weight index

^†^*p* value was derived from the Mann–Whitney test in data of continuous variables with abnormal distribution (M, Median; IQR, Interquartile Range). *p* value was derived from the Chi-square test or fisher’s exact test in data of categorical variables from IBS and healthy controls (n,%). *p < 0.05

### The 16 s rRNA data revealed distinct microbiome compositions in IBS patients

**Fig. 1:**
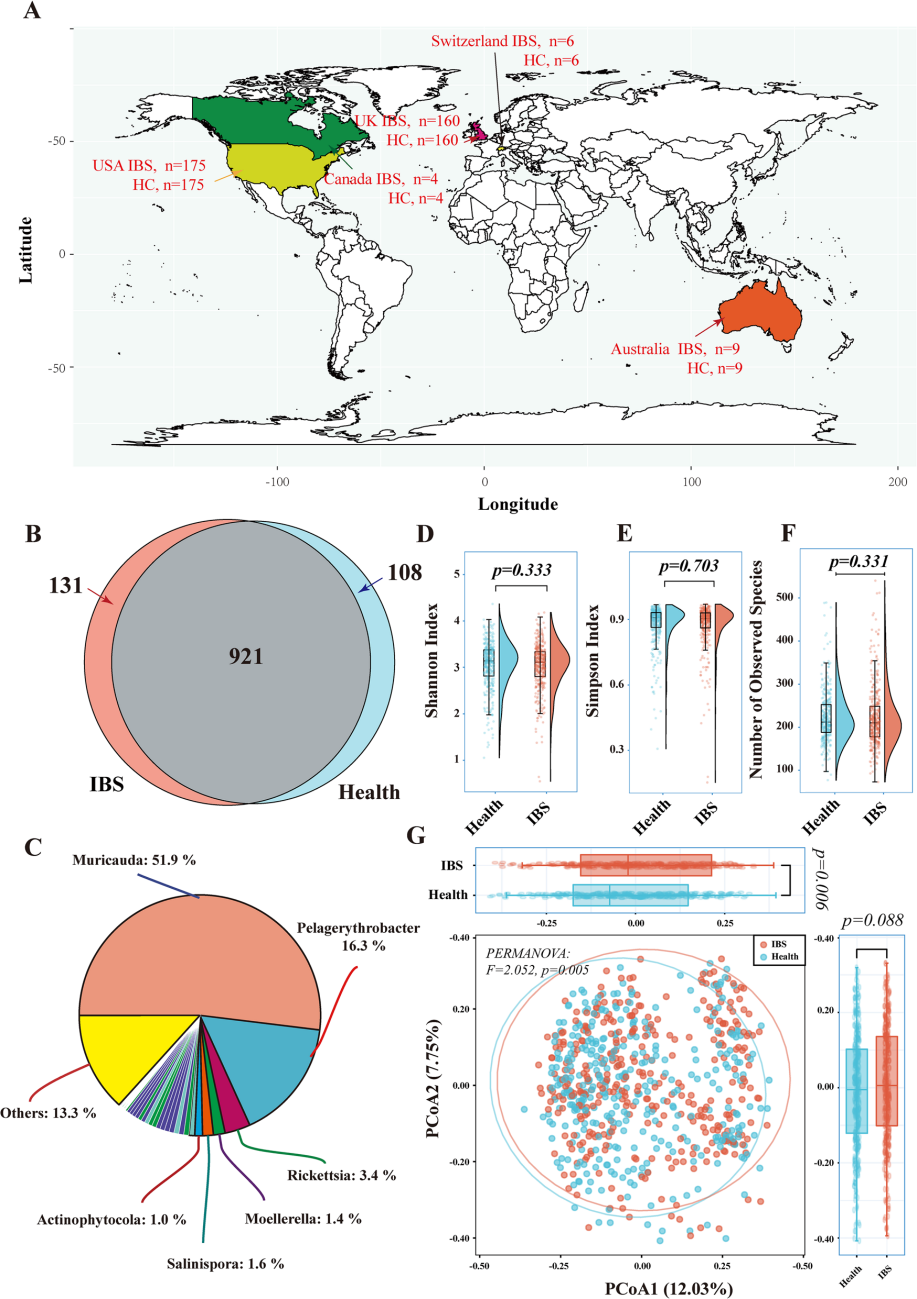
The geographic distribution of samples and microbial diversity evaluation. **A**: The geographic distribution of samples from five countries after the PSM procedure. **B**: The Venn diagram illustrating the number of species between IBS patients (pink) and healthy controls (light blue). **C:** The pie chart representing the composition of 131 IBS-exclusive genera identified from the Venn diagram. **D**–**F**: The rain-cloud plot showing the difference in alpha diversity between the IBS and healthy controls using the Shannon index (**D**), Simpson index (**E**) and Richness (**F**). **G**: PCoAs of Bray‒Curtis distances on the microbiota distributions. Each dot represents a patient with IBS or healthy controls. Points clustered in pink and light-blue eclipses represent the gut microbial composition of the IBS and controls, respectively. The boxplots around the PCoA plot represent the Bray-Curtis distances of Axis1 (the top boxplot) and Axis2 (the right-sided boxplot). The difference in alpha diversity index and Bray-Curtis distances of Axis1 and Axis2 was calculated using the Wilcoxon Rank Sum Test, and p < 0.05 was considered statistical significance. PERMANOVA test was performed, and p < 0.05 was considered statistical significance. IBS: irritable bowel syndrome; PCoAs: principal coordinate analyses

The final data included human stool samples from five countries (Fig. 1A). A total of 1,160 microbial genera (including 3,463 species) were identified in both IBS and healthy control groups. Within all the identified microbes, IBS and healthy control groups shared 941 genera, whereas 131 genera exclusively exited in the IBS group and 108 genera exclusively identified in the control groups (Fig. 1B, Additional file 2: Table S2). Among the 131 IBS-exclusive genera, the genera of *Muricauda* (phylum *Bacteroidetes*), *Pelagerythrobacter* (phylum *Proteobacteria*) and *Rickettsia* (phylum *Proteobacteria*) were the three most abundant microbes (Fig. 1C).

As for the alpha diversity, the richness of species, Shannon and Simpson index in the IBS groups presented no difference compared to healthy controls (Fig. 1D–F, Additional file 3: Table S3). When evaluating the beta diversity, we observed a significant difference in the Bray-Curtis distances in the Axis1 of PCoA (*p* = 0.006), but no significant difference in the Bray-Curtis distances in the Axis2 (*p* = 0.088) (Fig. 1G). The PERMANOVA test also revealed a significant dispersion difference (*p* = 0.005), indicating heterogeneous microbiota distributions between IBS and control groups.

**Fig. 2:**
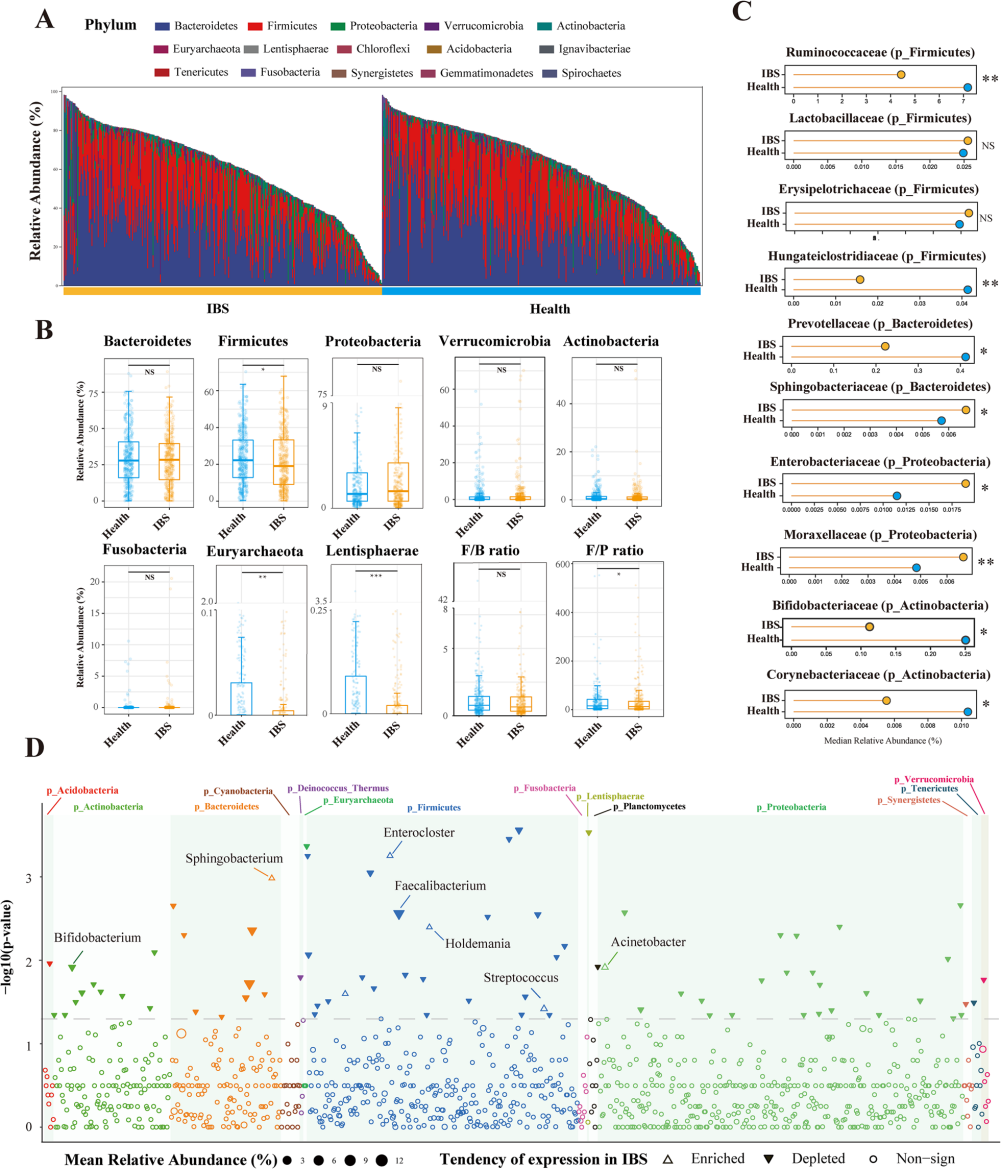
The differential analysis of microbiota compositions between IBS and healthy controls at the phylum, family and genus levels. **A**: The vertical bar chart presenting the microbiota compositions between IBS and healthy controls at the phylum level. The x-axis represents each sample and its group. The y-axis represents the relative abundance. **B**: Boxplots showing the relative abundance of eight phyla, *Firmicutes/Bacteroidetes* (F/B) ratio and *Firmicutes/Proteobacteria* (F/P) ratio between the IBS and healthy controls. **C**: Horizontal Lollipop plots representing the median relative abundance of significantly enriched or depleted families between the IBS and healthy controls. **D**: Manhattan plots showing the distributions of each genus identified in IBS and healthy individuals. Significantly-enriched genera are depicted as transparent triangles, significantly-depleted genera are presented as *inverted* solid triangles, and genera with no statistical significance are depicted as full circles. The dashed line corresponds to the p-value threshold of significance (p = 0.05). The color of each dot represents the different phylum affiliations. The size stands for their relative abundance. The green boxes are used to denote different phylum groups.The Wilcoxon Rank Sum Test was performed and *, **, *** stands for p-value < 0.01, 0.005 and 0.001, respectively)

At the phylum level, significantly decreased amounts of *Firmicutes* (*p* = 0.049), *Euryarchaeota* (*p* = 0.002), Cyanobacteria (*p* = 0.026), *Acidobacteria* (*p* = 0.049) and *Lentisphaerae* (*p* < 0.001) were observed in IBS groups compared with their healthy controls. The proportions of *Proteobacteria* and *Bacteroidetes* were increased in IBS groups, whereas the phyla *Bacteroidetes, Actinobacteria, Verrucomicrobia* and *Fusobacteria* were depleted, but none of the six researched a statistical significance (Additional file 4: Table S4). The *Firmicutes/Bacteroidetes* ratio showed no difference between the IBS and control groups (*p* = 0.130), whereas *Firmicutes/Proteobacteria* ratio was significantly decreased in the IBS group (*p* = 0.039) (Fig. 2A, B).

At the family level, the IBS group had significantly increased proportions of the family *Moraxellaceae* (phylum *Proteobacteria*) (*p* = 0.009), *Sphingobacteriaceae* (phylum *Bacteroidetes*) (*p* = 0.013) and *Enterobacteriaceae* (phylum *Proteobacteria*) (*p* = 0.046), whereas bacteria in the family *Ruminococcaceae* (phylum *Firmicutes*) (*p* = 0.001), *Bifidobacteriaceae* (phylum *Actinobacteria*) (*p* = 0.017) and the other 25 taxa are significantly decreased in the IBS group. The abundance of the family *Lactobacillaceae* (phylum *Firmicutes*) and *Erysipelotrichaceae* (phylum *Firmicutes*) were not significantly different in both groups. (Fig. 2C, Additional file 5: Table S5).

At the genus level, the genera *Enterocloster*, *Sphingobacterium*, *Holdemania, Acinetobacter*, *Bacillus* and *Streptococcus* were significantly enriched in the IBS cohort. The genera *Ruminococcus*, *Faecalibacterium*, *Bifidobacterium* and other 68 genera were significantly depleted in the IBS group (Fig. 2D, Additional file 6: Table S6).

At the species level, 219 species had significantly different proportions of abundance between the IBS and healthy groups, including 9 species significantly enriched (*Bacteroides fragilis*, *Blautia coccoides*, *Eggerthella lenta*, *Clostridium aldenense*, *Clostridium bolteae*, *Holdemania filiformis*, *Streptococcus oralis*, *Streptococcus mitis*, *Streptococcus suis*) and 210 species significantly depleted in the IBS groups. Of the 219 species, 12 belonged to the genera *Bifidobacterium*, 10 belonged to the genera *Clostridium*, 7 belonged to the genera *Streptococcus*, 6 belonged to the genera *Bacteroides* and 5 belonged to the genera *Lactobacillus* (Additional file 7: Table S7).

**Fig. 3:**
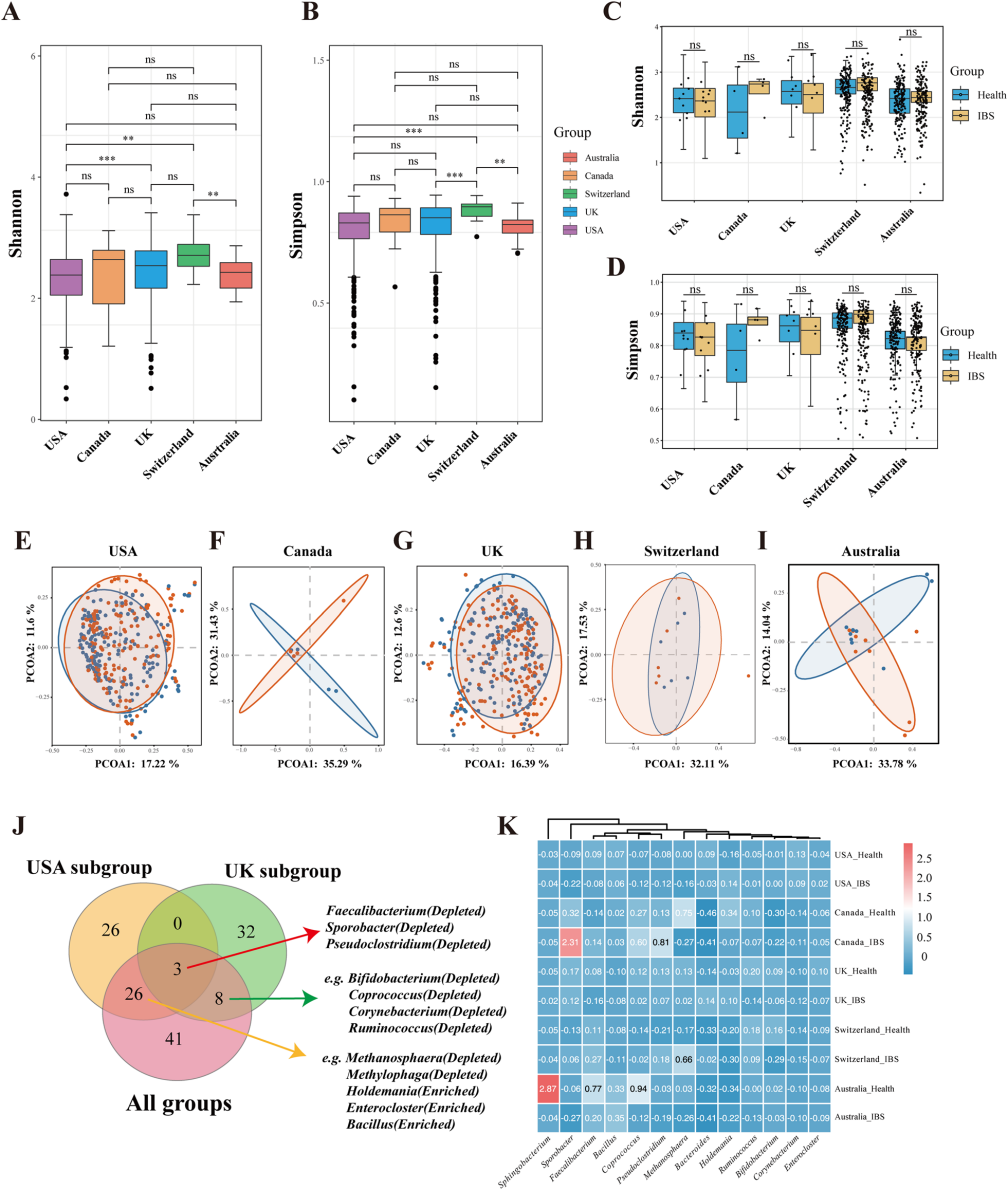
Subgroup analysis of the microbial distributions in different regions. **A**-**B**: Boxplots of the Shannon (A) and Simpson (B) index of samples belonging to five countries. **C**-**D**: Boxplots of the Shannon (C) and Simpson (D) index in groups of IBS and healthy controls in each country. **E**-**I**: PCoAs of Bray‒Curtis distances on the microbiota distributions in USA (E), Canada (F), UK (G), Switzerland (H), Australia (I). Each dot represents a patient with IBS or healthy controls. Points clustered in pink and blue eclipses represent the gut microbial composition of the IBS and controls, respectively. **J**: The Venn diagram illustrating the number of significantly enriched or depleted species between IBS and healthy controls in the whole cohorts, USA cohorts and UK cohorts. The IBS or control in parentheses means the group in which the species was enriched. The Wilcoxon Rank Sum Test was performed and *, **, *** stands for p-value < 0.01, 0.005 and 0.001, respectively). **K**: Heatmap visualization of the mean abundance in IBS patients and healthy controls based on regional differences. Each column represents one subgroup based on the IBS or control groups in different countries. Each row represents a genus. Values in each square represent the mean relative abundance in percentage (Log 10 transformed). The color scale was set based on the specific value of the mean relative abundance after the Log 10 transformation, with pink for relatively high abundance and blue for relatively low abundance

As geographical location may exhibit a great influence on the gut microbiota, we stratified data into subgroups by different regions. Figure 3A and 3B shows the comparisons of Shannon and Simpson indexes among countries. The Shannon index (3A) differs significantly between the USA and UK cohorts (*p* < 0.001), USA and Switzerland cohorts (*p* = 0.002) and Switzerland and Australia cohorts (*p* = 0.009). The Simpson index (3B) differs significantly between the USA and Switzerland cohorts (*p* < 0.001), UK and Switzerland cohorts (*p* < 0.001) and Switzerland and Australia cohorts (*p* = 0.001). However, the Shannon and Simpson indexes of the IBS and healthy controls had no significant difference within each country (Fig. 3C and D). The beta diversity (Fig. 3E-I) showed no significant difference in the USA, UK and Switzerland cohorts, whereas the Canada and Australia cohorts showed a statistical difference in the Bray–Curtis distances of Axis 2 in the PCoA analysis). Significantly-changed genera in each country cohort were shown in Additional file 9: Table S9. Among significantly-different genera, *Faecalibacterium*, *Sporobacter* and *Pseudoclostridium* were commonly depleted in IBS groups in samples of the USA, UK and all five countries. (Fig. 3J) The abundance change of several significant genera in the IBS group within each country was further presented in Fig. 3K. We observed that these genera might exist different tendencies of the proportion change in the IBS groups in different countries. *Faecalibacterium* was depleted in the IBS groups in the UK, USA and Australia, whereas it was slightly enriched in the IBS groups in the Canada and Switzerland cohorts. *Bifidobacterium* was depleted in the IBS groups in the *UK,* Switzerland and Australia, whereas it was slightly enriched in the USA and Canada areas.

### No strong correlations were found among microbes with age or BMI in IBS individuals

To further investigate whether the microbial abundance fluctuates with age or BMI, we applied the Spearman correlation test to find microbes potentially correlated with age and BMI. We observed no genera with relatively strong correlations (|ρ|> 0.4 and *p* < 0.05) with age or BMI, indicating that IBS individuals may have a relatively stable microbial composition unaffected by age or BMI (Additional file 8: Table S8).

### The abundance of several genera varied significantly in different IBS subtypes

**Fig. 4:**
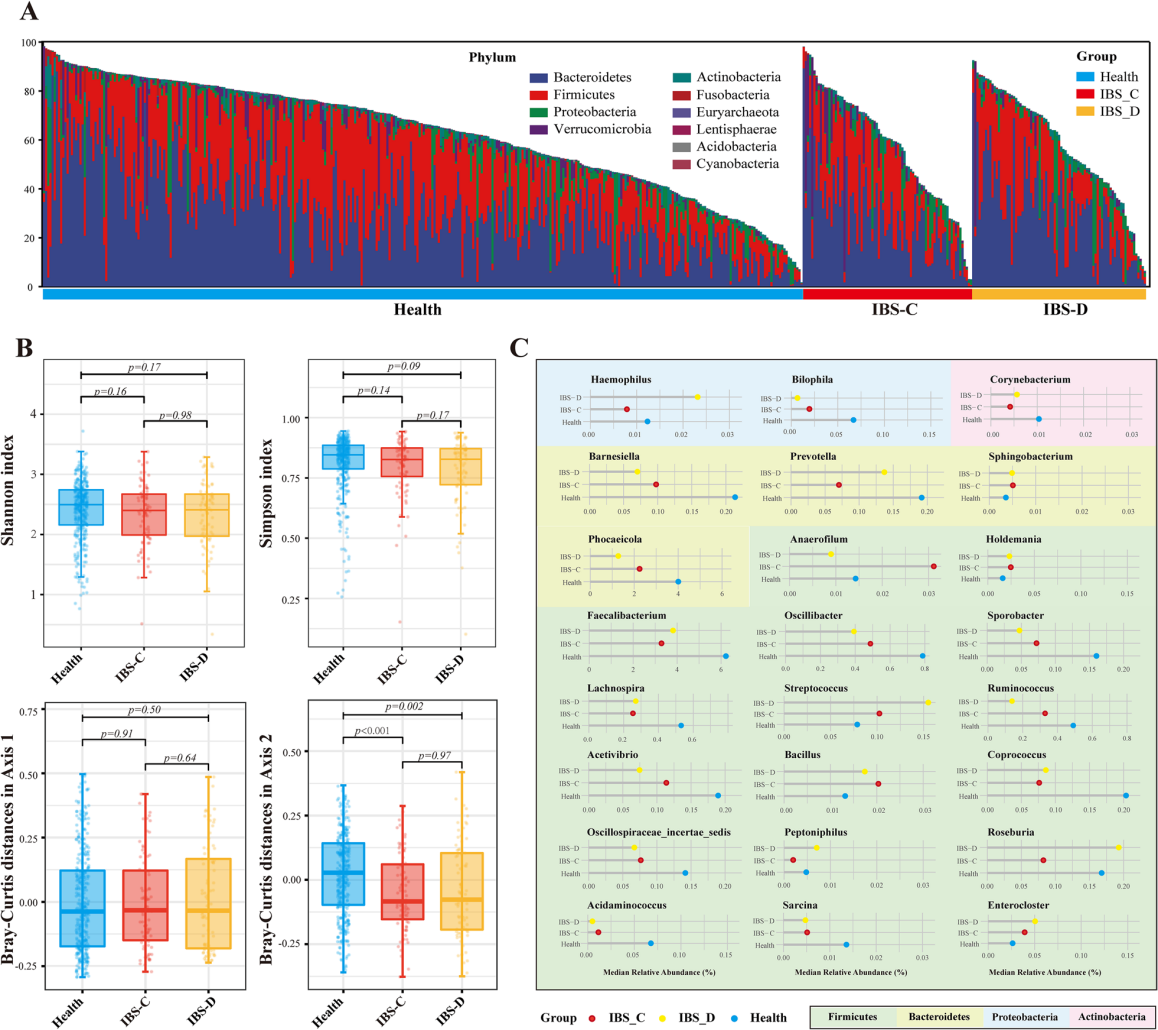
Subgroup analysis of the microbial compositions in different IBS subtypes. **A**: The vertical bar chart presenting the microbiota compositions among IBS with predominant constipation (IBS-C), predominant diarrhea (IBS-D) and healthy controls at the phylum level. **B**: Boxplots of the Shannon and Simpson index among the three groups. **C**. Horizontal Lollipop plots representing the median relative abundance of significantly enriched or depleted genera among the three groups. The background colors represent the different phyla they belong to. Kruskal-Wallis Test was performed and *, **, *** stands for p-value < 0.01, 0.005 and 0.001, respectively)

Considering the microbial composition in different IBS subtypes may vary, we selected the 16 s rRNA data of the IBS-C and IBS-D subtypes for further analysis. The differential analysis was performed in IBS-C, IBS-D and healthy controls to identify significantly different genera among the three groups. At the phylum levels, there was no overt difference in microbial compositions among groups (Fig. 4A). As for the microbial diversity, the Shannon and Simpson indices were not significantly different among groups, whereas the Bray–Curtis distances of Axis 2 in PCoA analysis showed a significant difference between Health and IBS-C (*p* < 0.001) and between Health and IBS-D (*p* = 0.002) (Fig. 4B). We identified 24 genera (Kruskal–Wallis test, *p* < 0.05) with significantly different abundance among the three groups. Compared with healthy individuals, the following genera had increased abundance proportions in both the IBS-C and IBS-D subgroups: *Streptococcus*, *Bacillus*, *Enterocloser*, *Sphingobacterium* and *Holdemania*. The genera *Faecalibacterium*, *Ruminococcus*, *Oscillibacter*, *Coprococcus* and the other 11 genera were depleted in both IBS subgroups. (Fig. 4C) When compared with healthy individuals, four genera showed a completely different trend of abundance change between the IBS-C and IBS-D. The genera of *Haemophilus*, *Peptoniphilus* and *Roseburia* were enriched in the IBS-D but depleted in IBS-C, whereas *Anaerofilum* was enriched in the IBS-C but depleted in IBS-D.

### Alternations of microbial genera in IBS patients with psychiatric disorders

**Fig. 5:**
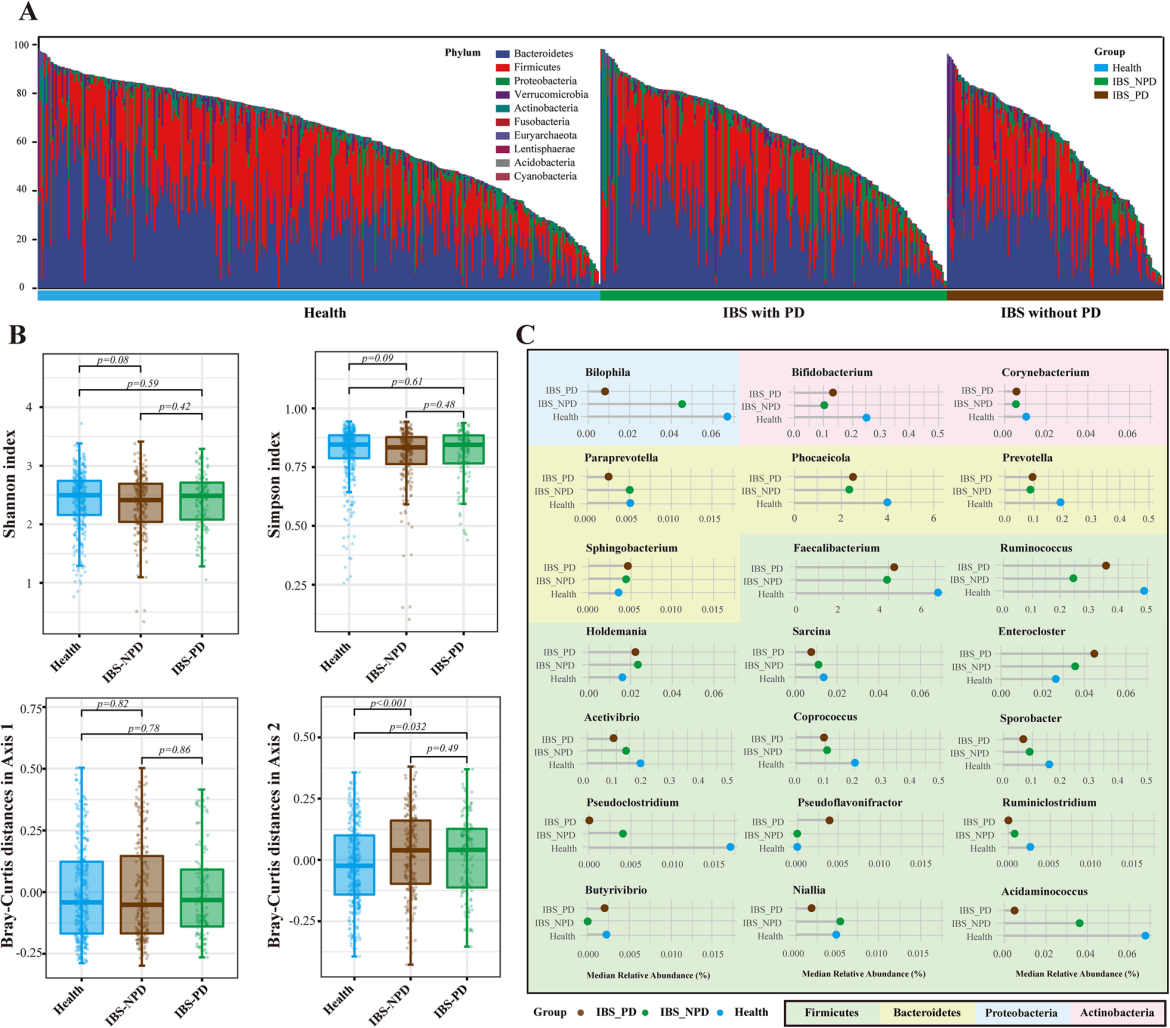
Subgroup analysis of the microbial compositions in IBS with or without psychiatric disorders. **A**: The vertical bar chart presenting the microbiota compositions among IBS with psychiatric disorders (IBS-PD), IBS without psychiatric disorders (IBS-NPD) and healthy controls at the phylum level. **B**: Boxplots of the Shannon and Simpson index among the three groups. **C**. Horizontal Lollipop plots representing the median relative abundance of significantly enriched or depleted genera among the three groups. The background colors represent the different phyla they belong to. Kruskal-Wallis Test was performed and *, **, *** stands for p-value < 0.01, 0.005 and 0.001, respectively)

Of all the 354 patients with IBS, 136 were diagnosed with psychiatric disorders. We specifically investigated the difference in microbial compositions among IBS patients with or without psychiatric disorders. At the phylum levels, there was no overt difference in microbial compositions among groups (Fig. 5A). The Shannon and Simpson indices were not significantly different among groups, whereas the Bray-Curtis distances of Axis 2 in PCoA analysis showed a significant difference between Health and IBS with psychiatric disorders (IBS-PD) (*p* = 0.032) and between Health and IBS without psychiatric disorders (IBS-NPD) (*p* < 0.001) (Fig. 5B). The Kruskal-Wallis test identified 21 genera with significantly different abundance among the three groups. The genera *Bilophila*, *Acidaminococcus, Pseudoclostridium* and the other five genera presented a step descent proportions of abundance in healthy, IBS-NPD and IBS-PD, whereas the genera *Enterocloster* had a stepped growth. (Fig. 5C).

### The co-occurrence network identified hub genera and species of IBS individuals

**Fig. 6:**
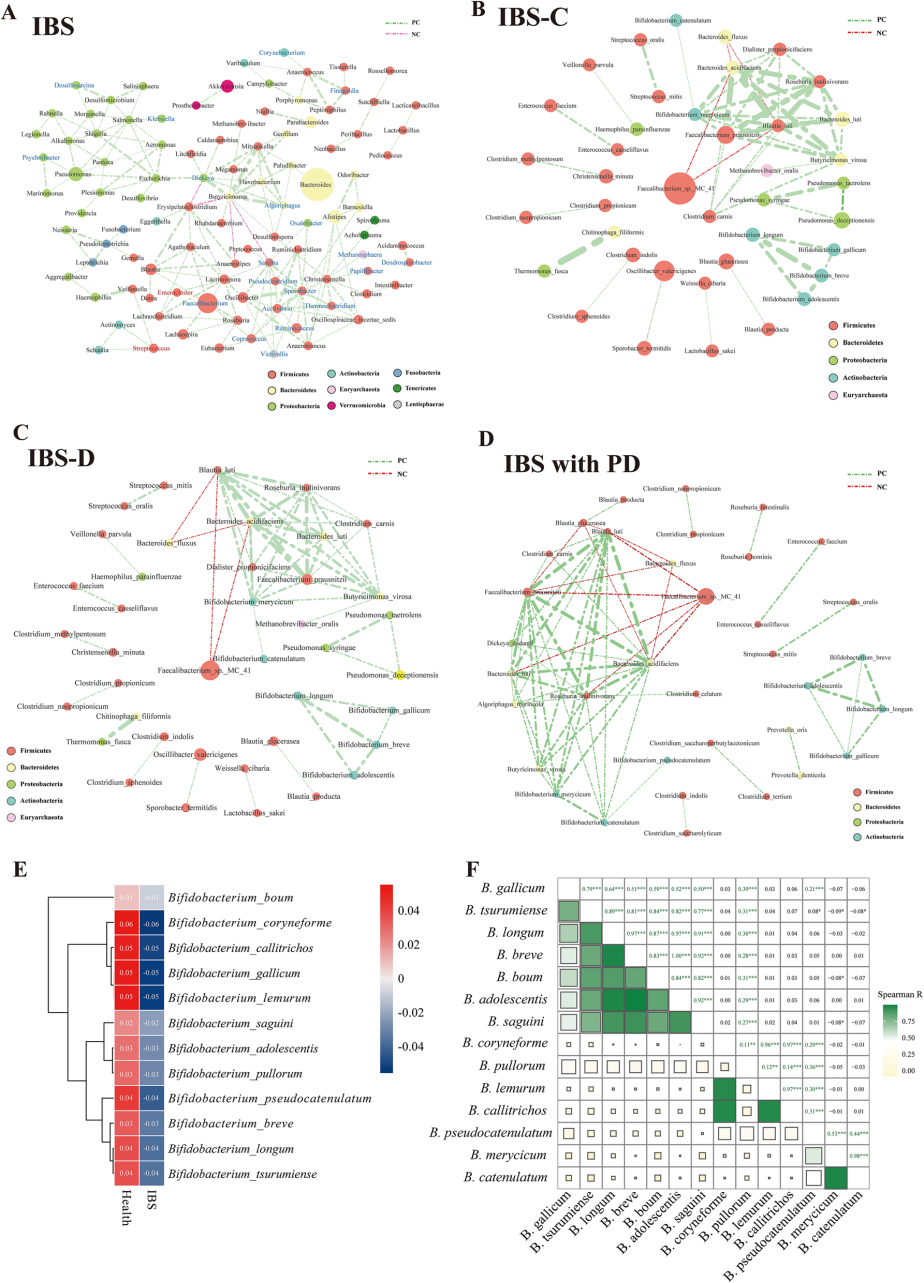
Co-occurrence network visualization of the microbial interactions in the IBS individuals. **A**: Co-occurrence network of microbes at the genera level. **B**–**D**: Co-occurrence network of microbes at the species level in IBS-C patients (**B**), IBS-D patients (**C**) and IBS with psychiatric disorders (**D**). The lines connecting nodes (edges) represent a positive (light green) or negative (red) co-occurrence relationship. The color of each dot represents the different taxonomic affiliations of the species (phylum level), the width of the edges reflects the absolute value of correlation coefficients. The size corresponds to their relative abundance. **E:** Heatmap visualization of the mean relative abundance of 12 significantly changed species belonging to the genera *Bifidobacterium* in IBS and healthy controls. Two columns represent the IBS and control, respectively. Each row represents one species of *Bifidobacterium*. Values were normalized by Z-scores. The color scale was set with red for relative high abundance (Z-scores > 0) and blue for the low ones (Z-scores < 0). The more weights of the absolute Z-scored-transformed abundance values, the deeper color of the squares. **F**: Heatmap matrix plot of Spearman's correlation coefficients (ρ) among different *Bifidobacterium* species. The absolute value of ρ is indicated by a color code explained in the legend. The green color indicates a positive correlation, whereas brown represents a negative one. The scale of a square is proportional to ρ2. Cells above the matrix diagonal refer to specific ρ values and their statistical significance (p-value). Significance levels p < 0.05, p < 0.01 and p < 0.001 are indicated by *, ** and ***, respectively, whereas p > 0.05 is presented p explicitly

The co-occurrence patterns among the IBS samples were explored using network inference based on strong (|ρ|> 0.8) and significant (*p* < 0.01) correlations. The network was composed of 102 nodes (microbes) and 171 edges. The entire network contained 11 modules, with 81 of the 102 genera occupied by the top five modules (modules 1–5). The top 3 ranked hub microbes were the genera *Butyricimonas*, *Christensenella* and *Pseudoclostridium* using the maximal clique centrality (MCC) method within the cytohubba (Fig. 6A).

To further explore the specific interactions of the microbial communities, we constructed three different networks at the species level in IBS subtypes of IBS-C, IBS-D and IBS with psychiatric disorders. We observed similar interaction modules in the three groups (Fig. 6B–D). By calculating the MCC, the top 10 hub species were identified in the three groups, respectively (Additional file 10: Table S10). We observed that six hub species were commonly shared by the three subgroups: *Faecalibacterium prausnitzii*, *Blautia lutim*, *Roseburia inulinivorans*, *Bacteroides acidifaciens*, *Bifidobacterium merycicum* and *Bacteroides luti.* We also observed a strongly positive interactions among species of the genera *Bifidobacterium* in all three networks.

### Strong positive interactions among the *Bifidobacterium* spp. in IBS individuals

To further investigate the interaction of species belonging to the genus *Bifidobacterium* (B)*,* we specifically analyzed the correlations of 12 *Bifidobacterium* species identified as significantly changed species in the IBS groups. These 12 species were all significantly depleted in IBS individuals (Fig. 6E). The Spearman analysis revealed that *B.longum, B.breve* and *B.adolescentis* had strong correlations (correlation coefficients (ρ) > 0.95, *p* < 0.001)*. *Some relatively lower abundant species were also strongly correlated with other *Bifidobacterium spp.* In these groups, *B.merycicum* was strongly correlated with *B.catenulatum* (ρ = 0.98, *p* < 0.001). *B.callitrichos* was strongly correlated with *B.coryneforme* (ρ = 0.97, *p* < 0.001) and *B.lemurum* (ρ = 0.97, *p* < 0.001). *B.coryneforme* was strongly correlated with *B.lemurum* (ρ = 0.96, *p* < 0.001) (Fig. 6F).

### Identification of microbial biomarkers for IBS individuals

**Fig. 7:**
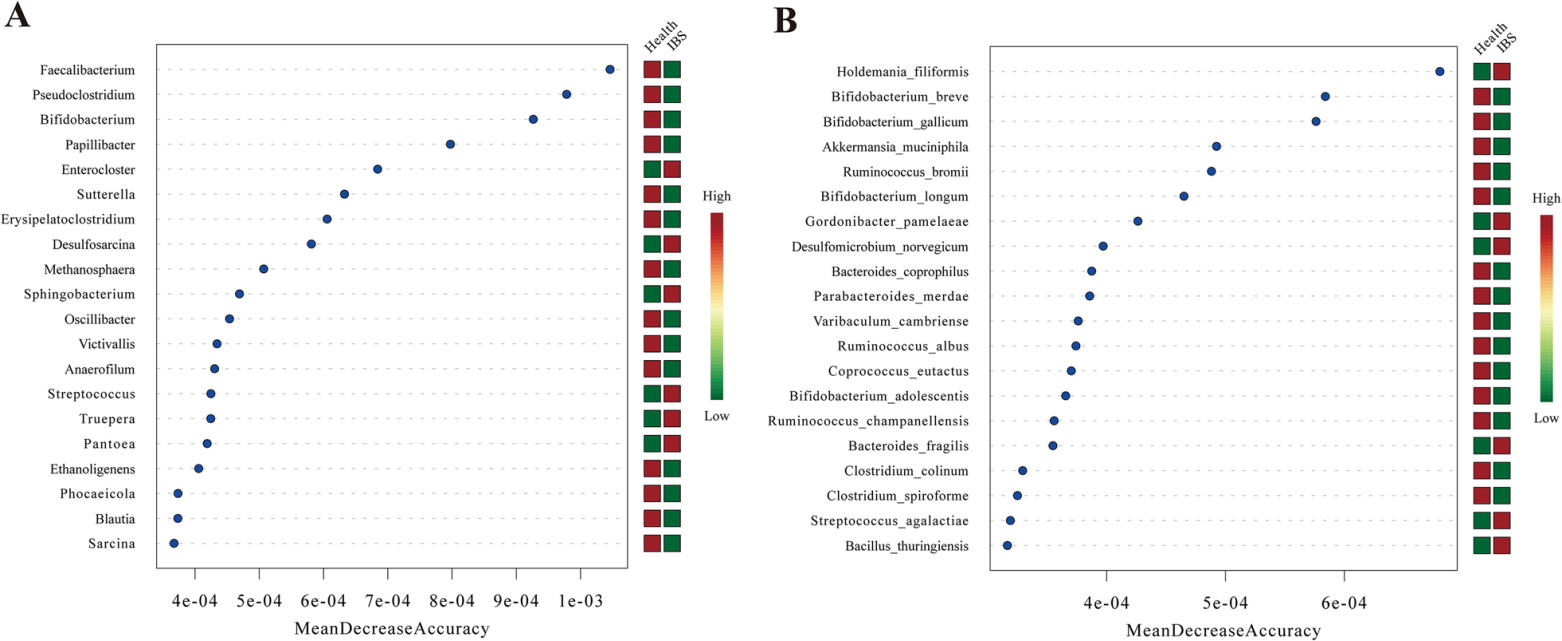
Random forest models.** A**: The random forest model of the top 20 ranked biomarkers identified at the genera level to distinguish IBS from healthy controls on their mean decrease scores of the optimal model performance. **B**: The random forest model of the top 20 ranked biomarkers identified at the species level. The red square on the right side of each genera represents the enrichment of this microbe in that group, whereas the green square represents the depletion of this microbe in that group

Finally, a random forest model was built and microorganisms with the top 20 values of the mean decrease accuracy (MDA) were identified for classifying IBS phenotype at the genus and species levels, respectively. The *Faecalibacterium, Pseudoclostridium* and *Bifidobacterium* were the top three genera (Fig. 7A) and the *Holdemania filiformis, Bifidobacterium breve* and *Bifidobacterium gallicum* were the top 3 species (Fig. 7B). Table 2 lists representative microbes that were significantly changed in IBS patients that might involve in IBS pathogenesis.

**Table 2:** Representative microbes that might involve in IBS pathogenesis

NCBI_taxa_ID	Rank	Taxon_name	Phylum	Significantly changed*	Trend in IBS	Top 20 in RF	Hub taxon
Taxon543	*Family*	*Enterobacteriaceae*	*Proteobacteria*	√	Enriched	–	–
Taxon1301	*genus*	*Streptococcus*	*Firmicutes*	√	Enriched	√	×
Taxon216851	*genus*	*Faecalibacterium*	*Firmicutes*	√	Depleted	√	×
Taxon1678	*genus*	*Bifidobacterium*	*Actinobacteria*	√	Depleted	√	×
Taxon2719313	*genus*	*Enterocloster*	*Firmicutes*	√	Enriched	√	×
Taxon35832	*genus*	*Bilophila*	*Proteobacteria*	√	Depleted	√	×
Taxon216816	*Species*	*Bifidobacterium_longum*	*Actinobacteria*	√	Depleted	√	×
Taxon1685	*Species*	*Bifidobacterium_breve*	*Actinobacteria*	√	Depleted	√	×
Taxon1680	*Species*	*Bifidobacterium_adolescentis*	*Actinobacteria*	√	Depleted	√	×
Taxon78342	*Species*	*Bifidobacterium_gallicum*	*Actinobacteria*	√	Depleted	√	×
Taxon1755626	*Species*	*Faecalibacterium_sp._MC_41*	*Firmicutes*	√	Depleted	×	√
Taxon853	*Species*	*Faecalibacterium_prausnitzii*	*Firmicutes*	√	Depleted	×	√
Taxon61171	*Species*	*Holdemania_filiformis*	*Firmicutes*	√	Enriched	√	×
Taxon89014	*Species*	*Blautia_luti*	*Firmicutes*	√	Depleted	×	√
Taxon360807	*Species*	*Roseburia_inulinivorans*	*Firmicutes*	√	Depleted	×	√

*IBS* irritable bowel syndrome, *NCBI* National Center for Biotechnology Information, *RF* random forest

^*^The relative abundance of IBS patients was compared with healthy controls, using the Mann–Whitney test. Significantly changed taxon was considered with a *p-*value < 0.05

## Discussion

To our knowledge, this is the first integrated microbial analysis using multicenter sequencing data in IBS individuals. The sample size is much larger than any of the previously reported studies. We specifically compared the microbial compositions in IBS patients with healthy controls and further identified hub microbes that may play a vital role in the whole bacterial community of IBS individuals.

First, this study highlighted several potentially harmful microbes with remarkably enriched abundance in IBS patients, including the family *Enterobacteriaceae* and the genus *Streptococcus*. The family *Enterobacteriaceae* belongs to the phylum *Proteobacteria which h*as been previously reported as one of the significantly enriched phyla in IBS patients [10, 20]. The *Enterobacteriaceae* family has been reported as a potentially harmful taxon because it contains several pathogenic bacteria (*Escherichia*, *Shigella*, *Campylobacter*, *Salmonella*, etc.) and thus might be associated with enteric infections [21]. This family has also been reported to be positively correlated with some inflammatory markers, such as interleukin-6 and interleukin-8 [22]. Thus, the enrichment of *Enterobacteriaceae* indicates that IBS patients may have enteric dysbacteriosis and potential inflammation, which promotes the growth of facultative, non-fastidious bacteria like *Enterobacteriaceae*. In addition to *Enterobacteriaceae*, the genus *Streptococcus,* as one of the members in aerobe groups, was also a potentially harmful microbe of IBS individuals. This finding was consistent with another study conducted by Rajilić-Stojanović et al. [23] which reported significant enrichment of *Streptococcus* in IBS patients. We also observed that *Streptococcus* was remarkably enriched in both IBS-C and IBS-D subgroups, indicating that both diarrhea and constipation symptoms may be associated with underlying intestinal dysbacteriosis. *Streptococcus oralis*, *Streptococcus mitis* and *Streptococcus suis* were three IBS-enriched species detected in our study. The three species have been reported as the most frequently identified oral colonized pathogens [24]. Our findings suggested that patients with IBS might also have altered oral microbiota, possibly due to different dietary structures and oral hygiene habits.

Furthermore, we emphasized the importance of the genera *Faecalibacterium* and *Bifidobacterium* as two potentially protective taxa in IBS individuals. *Faecalibacterium prausnitzii* is a butyrate-producing microbe with anti-inflammation efficacy by mediating the expression of interleukin-17 [25], and it can also enhance the integrity of gastrointestinal barriers [26, 27]. Our study not only confirmed the significantly decreased abundance of *Faecalibacterium* reported in previous observational studies [11, 23, 28, 29] but also identified that *Faecalibacterium prausnitzii* was a hub microbe in the whole microorganism community of IBS groups. In the co-occurrence network, we observed strong positive correlations of *Faecalibacterium prausnitzii* with other butyrate-producing bacteria, such as *Butyricimonas virosa, Roseburia inulinivorans* and *Bacteroides acidifaciens.* This finding indicates that the *Faecalibacterium prausnitzii* is a central hub highly connected to other commensally-beneficial microorganisms that may serve as protecting agents in controlling IBS pathogenesis. Clinical trials are needed to further evaluate the potential values of *Faecalibacterium prausnitzii* supplements in treating patients with IBS. Another finding of our study is the strong positive correlations among *Bifidobacterium* species, especially the interactions of *B. longum, B.breve* and *B. adolescentis.* The oral administrations of *B. longum* and *B. breve *have been confirmed by several clinical trials [30–32] with promising treatment efficacy on IBS patients, while *B. adolescentis* is a diet-sensitive microbe that can be supplied by certain food to relieve IBS symptoms [33]. Our findings suggest that supplementing one of the above *Bifidobacterium* species may promote the growth of others in the whole *Bifidobacterium* community. Thus, more trials are needed to evaluate the clinical efficacy of transplanting dominant *Bifidobacterium* species in IBS patients. Future studies are also needed to investigate whether such interactions among *Bifidobacterium* species may promote the treatment efficacy of *Bifidobacterium* probiotics in IBS patients.

It is reported that IBS can affect the development and pathology of neurodegenerative and neuropsychiatric diseases [34]. However, this aspect is less well-studied. Our findings suggest that the genera *Bilophila* and *Enterocloster* may be potential biomarkers of IBS patients along with psychiatric disorders. However, no direct evidence has demonstrated the possible association between IBS and these two microbes. Therefore, more relevant studies are needed to further validate the potential role of these two microbes in the development of IBS and psychiatric disorders through the gut-brain axis.

Our study has also raised some concerns that should be further addressed. First, we observed some conflicting results during microbial differential analysis. For instance, several studies reported a significant increase in the family *Lactobacillaceae*, but we observed no statistical difference in this family in IBS and controls. Conflicting results were also seen in the phyla *Firmicutes*, *Bacteroidetes* and the genus *Ruminococcus*, etc. This issue has been mentioned in several previous studies [11, 23]. The possible explanation is that microbial compositions may vary in different IBS subtypes, as we found that the genera of *Haemophilus*, *Peptoniphilus*, *Roseburia* and *Anaerofilum* presented completely different trends of abundance change between IBS-C and IBS-D. In addition, we observed relatively low MDA values in all microbial biomarkers in the random-forest model, indicating that the gut microbiota alone could not precisely distinguish IBS patients from healthy controls. Therefore, it is necessary to establish a comprehensive predictive model for IBS by integrating multiple variables, including the intestinal microorganisms, essential metabolites and clinicopathological characteristics, rather than using IBS-specific microbes alone.

Our study has several limitations.First, the sample size is not uniform across the regions. We were unable to enroll more samples in the cohorts of Canada, Switzerland and Australia, as few data were available regarding IBS patients from these regions in the GMrepo database. Second, we were unable to access the detailed data on clinical characteristics, such as IBS symptom scores, diet structure and a history of previous intestinal infections, due to the unavailability of relevant information provided in the GMrepo database. Thus, we only included age and BMI as environmental factors to analyze their correlations in IBS patients. Third, similar to most previous studies, we did not use corrected p-values (FDR) after multiple comparisons because many significantly-differentiated species presented increased false positive rates (> 0.1) after p-correction. Based on the p-values reported previously, it may also be the reason for most studies without performing p corrections.

In conclusion, this study provides quantitative analysis and visualization of the interaction between the gut microbiota and IBS using multicenter amplicon sequencing data. The identification of key species should be further validated to evaluate their causal relationships with the pathogenesis of IBS.

## Supplementary Information

**Additional file 1: Table S1.** The baseline information of the 708 qualified samples from the GMrepo database.

**Additional file 2: Table S2.** Classification of the IBS-exclusive genera, health-exclusive genera and commonly identified genera.

**Additional file 3: Table S3.** Data of Alpha diversity.

**Additional file 4: Table S4.** Differential analysis of microbial compositions between IBS and healthy controls at the phylum level.

**Additional file 5: Table S5.** Differential analysis of microbial compositions between IBS and healthy controls at the family level.

**Additional file 6: Table S6.** Differential analysis of microbial compositions between IBS and healthy controls at the genera level.

**Additional file 7: Table S7.** Differential analysis of microbial compositions between IBS and healthy controls at the species level.

**Additional file 8: Table S8.** The Spearman correlation of gut microbiota and age/BMI in IBS individuals.

**Additional file 9: Table S9.** Significantly-changed genera in each sub-regional group.

**Additional file 10: Table S10.** The top 10 hub species identified in each microbial co-occurrence network.
